# Circadian rhythms in major depressive disorder: mechanistic insights and therapeutic frontiers

**DOI:** 10.1080/07853890.2026.2671594

**Published:** 2026-06-03

**Authors:** Saboor Saeed, Ruiqi Sang, Lim Zhixin, Huaizhi Wang, Le Xu, Xuhong Zhang, Shaohua Hu

**Affiliations:** a Department of Psychiatry, The First Affiliated Hospital, Zhejiang University School of Medicine, Hangzhou, China; b Nanhu Brain-computer Interface institute, Hangzhou, China; c Liangzhu Laboratory, Zhejiang University School of Medicine, Hangzhou, China; d The Zhejiang Key Laboratory of Precision Psychiatry, Hangzhou, China; e Beijing Key Laboratory of Mental Disorders, National Clinical Research Center for Mental Disorders and National Center for Mental Disorders, Beijing Anding Hospital, Capital Medical University, Beijing, China; f The State Key Lab of Brain-Machine Intelligence, Zhejiang University, Hangzhou, China; g MOE Frontier Science Center for Brain Science and Brain-Machine Integration, Zhejiang University School of Medicine, Hangzhou, China; h Brain Research Institute of Zhejiang University, Hangzhou, China; i Zhejiang Engineering Center for Mathematical Mental Health, Hangzhou, China; j Zhejiang University, School of medicine, Hangzhou, China

**Keywords:** Circadian rhythms, major depressive disorder, clock genes, chronotherapy, sleep-wake cycle

## Abstract

**Background:**

Major Depressive Disorder (MDD) has emerged as a leading cause of disability worldwide, affecting over 264 million people. Recent evidence reveals that disruption of circadian rhythms may be fundamental to MDD pathophysiology, opening new avenues for therapeutic intervention.

**Methods:**

This review synthesizes current understanding of the intricate relationship between circadian system disruption and MDD, highlighting molecular mechanisms and clinical implications. We examine evidence from genetic studies, clinical observations, and therapeutic trials.

**Results:**

Patients with MDD exhibit profound alterations in circadian-regulated processes, including sleep-wake cycles, diurnal mood patterns, and metabolic functions. Genetic studies have identified variants in core clock genes, particularly CLOCK, TIMELESS, and CRY1, that correlate with both circadian disruption and MDD susceptibility. These genetic insights, combined with evidence of dysregulated hypothalamus-pituitary-adrenal axis function and abnormal melatonin signaling, suggest that circadian dysfunction may be causal in MDD pathogenesis rather than merely symptomatic.

**Conclusions:**

Emerging chronotherapeutic approaches, such as light therapy, sleep interventions, and targeted pharmacology, show significant potential for improving depressive symptoms. Personalized circadian-based treatments, guided by genetic and molecular markers, could transform MDD care. Advancing our understanding of the circadian-depression connection offers a promising path to revolutionizing treatment strategies.

## GRAPHICAL ABSTRACT

**Figure:**
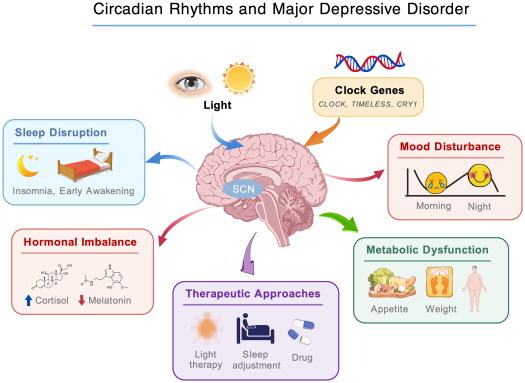

## Introduction

Major Depressive Disorder (MDD) [1] is a severe mental illness that has witnessed a nearly 50% surge in cases globally over the past 30 years, affecting more than 264 million people [2,3]. MDD not only significantly diminishes the quality of life for patients but also ranks as one of the leading causes of disability worldwide stemming from both the direct burden of mental symptoms and the disorder’s high prevalence [4] and mortality rates. Additionally, it imposes a considerable economic burden on society through loss of productivity [5,6]. Clinically, MDD is characterized by diverse symptoms, including but not limited to depressed mood, cognitive impairment, diminished volition, psychomotor disturbances, and various somatic symptoms. These complex manifestations reflect the intricate pathophysiology of MDD, which involves the relationship of multiple factors. Understanding these mechanisms is vital for developing effective treatments, improving patient quality of life, and alleviating socioeconomic burdens.

Research has shown that family history is strongly associated with MDD risk [7,8]. Other contributing factors include alterations in inflammatory markers and stress hormone expression [9,10], and abnormalities in neurotransmitters such as serotonin, norepinephrine, dopamine, glutamate, and gamma-aminobutyric acid [11–13]. Despite these findings, the pathophysiological mechanisms of MDD remain inadequately elucidated. In recent years, the relationship between circadian rhythms and MDD has garnered increasing attention. Circadian rhythms refer to the natural, approximately 24-hour cycles of physiological and behavioral processes in organisms [14], which are crucial for regulating sleep-wake cycles, hormonal balance, metabolic functions, and mood. Studies suggest that disruptions in circadian rhythms may play a role in the pathogenesis of MDD and that restoring normal circadian rhythms could help prevent and treat the disorder [15].

Recent studies in 2024 have provided compelling evidence linking circadian dysfunction to MDD through genetic, experimental, and longitudinal research. Large-scale population analyses have identified sex-specific associations between circadian-related genes and depressive symptoms [16], implicating metabolic, inflammatory, and neuroplasticity pathways Preclinical models further demonstrate dysregulation of core clock genes (Bmal1, Per1, Per2) in treatment-resistant depression, supporting a causal role of circadian disruption [17]. Additionally, wearable-based longitudinal studies reveal bidirectional relationships between sleep-wake cycles and circadian rhythms and mood, highlighting circadian regulation as a promising therapeutic target [18].

This review explores the complex relationship between circadian rhythm disruption and MDD, summarizing their interactions and underlying biological mechanisms. Additionally, it discusses therapeutic strategies targeting circadian rhythms and their potential effectiveness in regulating sleep-wake cycles and alleviating depressive symptoms. The importance of researching this relationship is emphasized, with the aim of developing more effective prevention and treatment strategies to enhance patients’ quality of life.

## Methodology

This article is narrative review synthesizing evidence on circadian rhythm disruption in major depressive disorder. Relevant literature was identified through searches of PubMed, Web of Science, and Scopus using keywords related to circadian biology, sleep, light exposure, and depression. Peer-reviewed experimental, translational, and clinical studies published in English were considered.

Given the narrative design of this review, no protocol preregistration (e.g. PROSPERO) was undertaken, and no formal risk-of-bias assessment was performed. Instead, methodological limitations and potential sources of bias within the included studies are discussed qualitatively where relevant. Evidence was integrated using a thematic and interpretative synthesis. Ethical approval was not required, as only previously published data were analyzed.

## Conceptual framework and scope of this review

This narrative review synthesizes current evidence on circadian dysfunction in MDD using a phenotype-focused and mechanism-based perspective. Rather than reiterating general principles of circadian biology, we concentrate on how specific circadian features such as shifts in timing, changes in rhythm amplitude, and altered sensitivity to light differ across depressive subtypes and relate to clinical symptoms and treatment response. By integrating findings from molecular, neuroendocrine, behavioral, and clinical studies, this review aims to clarify the clinical relevance of circadian disturbances and highlight their implications for targeted chronotherapeutic approaches.

## Overview of circadian rhythms

**Figure 1.**
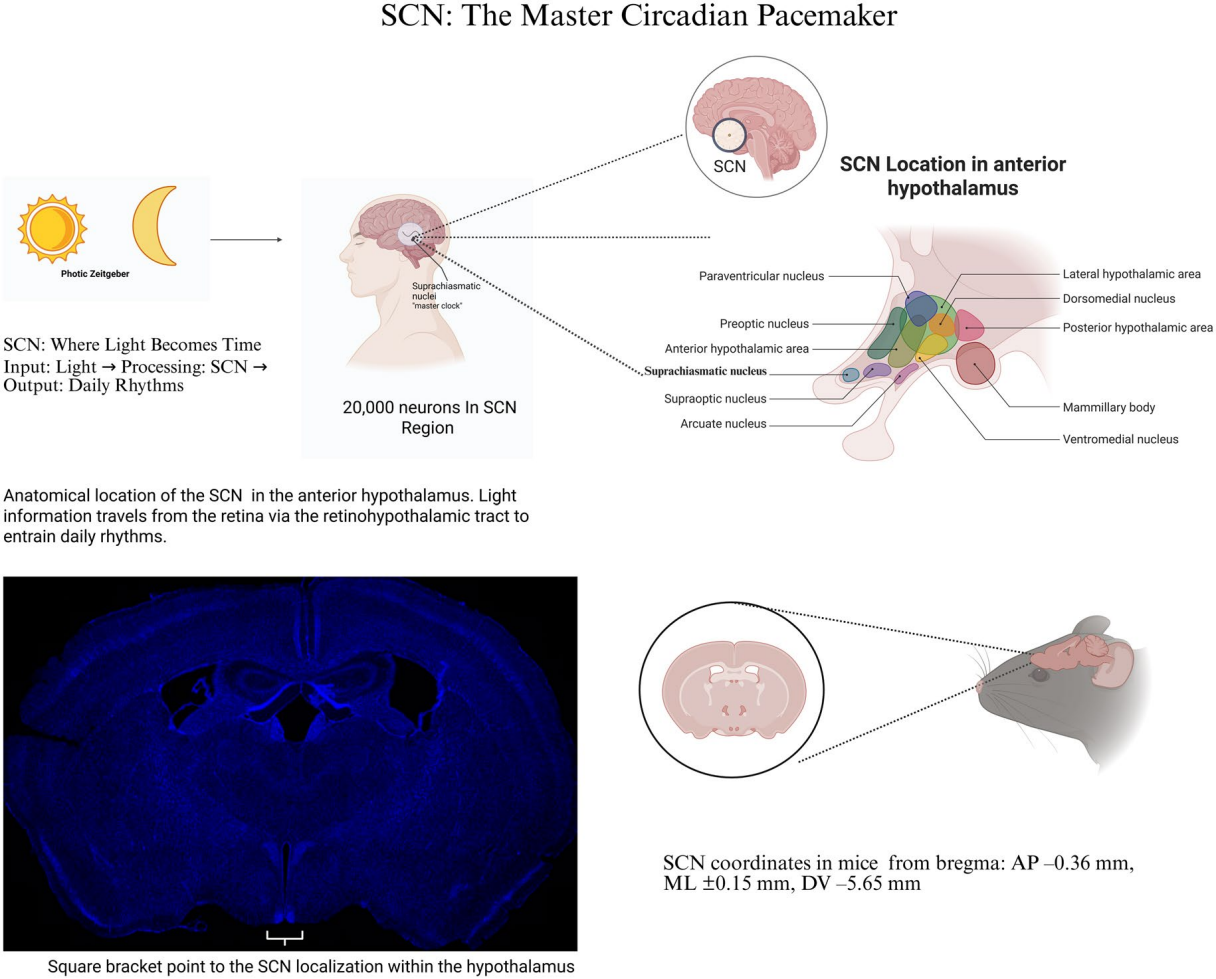
SCN: The master circadian pacemaker and its role in daily rhythms. SCN, located in the anterior hypothalamus, is the master pacemaker that regulates circadian rhythms by processing light signals through the retinohypothalamic tract and synchronizing biological rhythms with environmental light-dark cycles. Comprising approximately 20,000 neurons, the SCN relies on key neurotransmitters such as GABA, VIP, and AVP to coordinate daily rhythms across physiological and behavioral systems. In rodent models, the SCN’s precise anatomical location has been identified as −0.36 mm AP, ±0.15 mm ML, and −5.65 mm DV relative to Bregma, offering a valuable framework for translational research. These insights into the SCN’s structure and function highlight its critical role in circadian biology and its potential relevance in understanding mood disorders such as MDD.Additionally, a DAPI-stained coronal section illustrates the anatomical location of the SCN in the mouse brain, providing visual confirmation of the nucleus’s position for translational research purposes.

The circadian rhythm system operates based on three core components: input signals, internal oscillators, and output pathways [19]. This fundamental design principle is evident at both the systems biology and cellular biology levels. The biological clock serves as the intrinsic molecular mechanism of circadian rhythms and is divided into central and peripheral clocks, with light acting as a key input signal to calibrate and adjust the clock [20]. The suprachiasmatic nucleus (SCN) in the hypothalamus functions as the central clock, coordinating activities in other brain regions and peripheral tissues *via* neural signaling and hormone release to maintain circadian rhythm consistency and adaptability [21]. SCN, acts as the master pacemaker that regulates circadian rhythms in both humans and rodents (Figure 1). It processes light information received from the retina *via* the retinohypothalamic tract, converting environmental light cues into daily biological rhythms that govern various physiological and behavioral processes. With approximately 20,000 neurons, the SCN relies on key neurotransmitters such as GABA, VIP, and AVP to maintain its rhythmicity and coordinate downstream circadian outputs. As a central regulator of the circadian system, the SCN is a pivotal focus for exploring the link between circadian dysfunction and mood disorders like MDD. This makes it a critical subject of interest in translational research aiming to develop novel circadian-based therapeutic strategies Clock genes are a specific group of genes that form transcription-translation feedback loops (TTFLs) to sustain and regulate circadian rhythms.

While the SCN is the master regulator of circadian rhythms, light also exerts direct, non-circadian effects on mood through intrinsically photosensitive retinal ganglion cells (ipRGCs) [22]. These cells contain the photopigment melanopsin and project not only to the SCN but also directly to brain regions involved in emotional processing, such as the amygdala, lateral habenula, and the ventromedial prefrontal cortex (vmPFC) [23]. This direct pathway explains how light exposure can acutely modulate mood and cognitive function independently of the biological clock.

**Figure 2.**
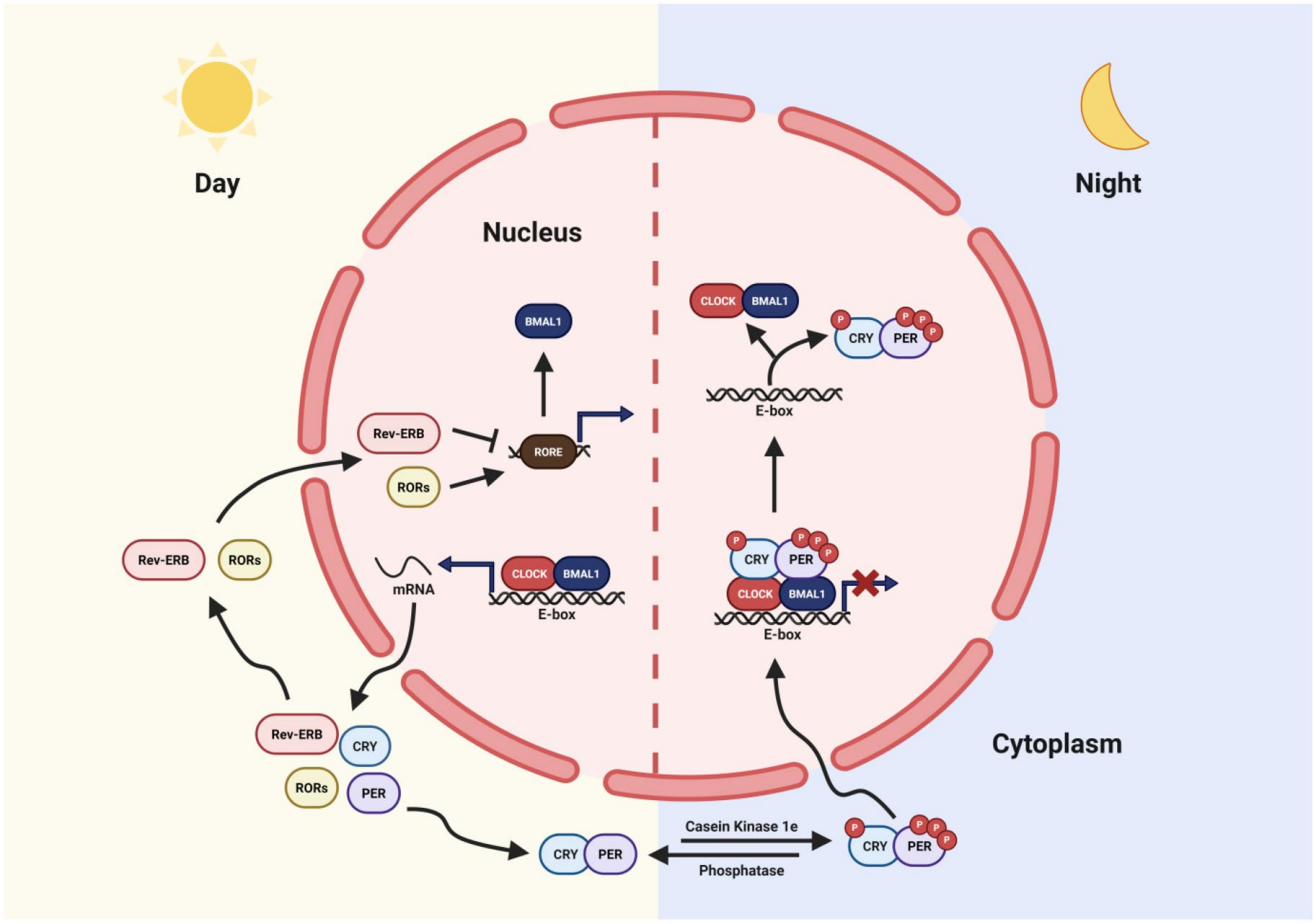
Transcription-translation feedback loop of core circadian clock genes within cells. During the daytime, CLOCK and BMAL1 form a heterodimer that binds directly to E-box enhancer elements, driving the transcriptional activation of genes encoding PER1, PER2, PER3, CRY1, and CRY2. At night, PER and CRY proteins form heterodimers and translocate into the nucleus, where they interact with the CLOCK-BMAL1 complex to repress their transcriptional activity. Subsequently, PER and CRY proteins undergo ubiquitination mediated by E3 ubiquitin ligases, leading to their progressive degradation. This relieves the negative feedback inhibition, allowing the transcriptional activity of CLOCK and BMAL1 to resume, initiating a new circadian cycle the following day.

In mammals, TTFLs generate and maintain circadian rhythms through a series of transcriptional and translational activities. CLOCK and BMAL1 proteins form heterodimers [24,25] that bind to E-box enhancer elements during the day, driving the expression of genes encoding circadian clock proteins PER1, PER2, PER3, CRY1, and CRY2 at night, PER and CRY proteins form heterodimers that translocate to the nucleus and interact with CLOCK and BMAL1, inhibiting their transcriptional activity [26]. Meanwhile, PER1, PER2, CRY1, and CRY2 proteins are degraded *via* ubiquitination, involving specific E3 ubiquitin ligases. As the negative feedback loop is gradually lifted, CLOCK and BMAL1 transcriptional activity resumes, initiating the next cycle. This periodic process spans approximately 24 h (Figure 2). Nuclear receptor families stabilize core loops at different times and regulate the expression of output genes. For instance, REV-ERBα and REV-ERBβ (encoded by NR1D1 and NR1D2) and orphan receptors RORα, RORβ, and RORγ are direct targets of CLOCK-BMAL1 [27,28]. These receptors competitively bind to RORE elements, antagonistically regulating BMAL1 and influencing the phase dynamics of BMAL1 and PER2. A third transcriptional loop includes DBP, TEF, and HLF proteins and their inhibitor NFIL3, which competitively bind to D-boxes under the regulation of the CLOCK-BMAL1 or REV-ERB-ROR loops [21]. The Role of SCN is also widely used in rodent’s model.

Clock genes exhibit pronounced tissue specificity, reflecting the unique physiological roles of distinct tissues and their differential adaptation to environmental cues [29,30]. Studies have demonstrated that approximately 43% of genes across 12 mouse tissues exhibit oscillatory expression in at least one tissue, underscoring the pervasive influence of circadian rhythms on gene regulation at the tissue level [31]. Similarly, transcriptomic analysis in 64 tissues and brain regions of male baboons revealed that over 80% of clock genes encode proteins with rhythmic expression in at least one tissue, highlighting the conserved and universal role of circadian control in gene expression [32]. These findings emphasize the intricate regulatory networks that coordinate gene expression to enable precise temporal execution of essential physiological processes.

The regulation of circadian clock genes represents a complex, multilayered process involving core circadian regulators, secondary transcription factors, cell-type-specific co-regulators, and epigenetic modulators [19]. The mechanisms underlying tissue-specific circadian rhythms are gradually being elucidated [33,34]. For instance, hepatocyte nuclear factor 4α, rhythmically expressed in the mouse liver, modulates circadian rhythms by repressing CLOCK-BMAL1 activity, thereby contributing to tissue-specific regulation [33]. In pancreatic islet cells, circadian transcriptomic analyses have identified genes critical for the temporal regulation of insulin secretion, further underscoring the functional importance of clock gene specificity [34].

Epigenetic modifications, including histone modifications, chromatin remodeling, and topological chromatin organization, significantly influence the regulation of E-box and RORE elements, further fine-tuning circadian gene expression [19,35]. These findings are expanding our understanding of how circadian clocks orchestrate tissue-specific gene expression programs. By elucidating the molecular underpinnings of these regulatory mechanisms, this research provides a foundation for the development of innovative therapeutic strategies targeting circadian disruptions in disease states.

## The link between circadian rhythms and major depressive disorder

Circadian rhythm disruptions are intricately connected to the pathophysiological alterations observed in MDD.In patients with MDD, these disruptions manifest as sleep-wake cycle disturbances, diurnal mood variation, seasonal affective patterns, altered feeding behaviors, and metabolic dysregulation. These clinical features are closely tied to abnormalities in neurotransmitter synthesis and release, dysregulated melatonin signaling, and impaired hypothalamus-pituitary-adrenal (HPA) axis function. Elucidating the relationship between circadian rhythm disturbances and the clinical manifestations of MDD is pivotal for advancing our understanding of its underlying pathophysiology. Such insights have profound implications for refining diagnostic approaches, optimizing therapeutic strategies, and devising preventive measures, ultimately contributing to more effective management of this debilitating condition.

## Disruption of sleep-wake mechanisms

Dysregulation of sleep-wake mechanisms is a key characteristic of circadian rhythm disturbances. Research has shown that sleep-wake disruptions are very common among individuals with MDD, impacting 50–90% of patients. Diurnal mood variation (DMV) is a hallmark of MDD, traditionally characterized by “morning-worst” symptoms. However, recent evidence suggests that “evening-worst” patterns are also prevalent, particularly in patients with an evening chronotype [36]. The mechanism of DMV involves a complex interaction between the circadian phase and homeostatic sleep pressure (Process S). In many patients, the antidepressant effect of sleep deprivation is thought to arise from the acute resetting of this interaction, leading to rapid mood improvement [18]. These disruptions can present as difficulties in falling asleep, early morning awakenings, excessive day time sleepiness, frequent nighttime awakenings, and nightmares [37]. These symptoms significantly hinder daily functioning and psychological well-being and are widely acknowledged as essential diagnostic criteria for MDD. Compared to healthy individuals, patients with MDD frequently display abnormal sleep patterns and altered sleep architecture. These changes include shorter rapid eye movement (REM) sleep latency, a longer first REM period, increased eye movement density during REM sleep, reduced slow-wave sleep (SWS), and decreased total sleep time [38–40]. Importantly, SWS activity during non-REM sleep, a crucial indicator of sleep homeostasis, is often lower in MDD patients [41]. Evidence indicates that poor sleep quality can exacerbate depressive symptoms [42], with sleep duration significantly correlating with depressive symptoms over time [43]. Furthermore, improvements in sleep are often predictive of a reduction in depressive symptoms [44]^.^ These findings underscore the importance of enhancing sleep quality and realigning sleep rhythms as a central strategy in the treatment of MDD. While sleep disturbances are among the common associated features of MDD, affecting the majority of patients, they are considered a core associated feature rather than an essential requirement for diagnosis according to DSM-5 criteria.

## Association between circadian clock gene variations and sleep disturbances in MDD

Sleep disturbances in patients with MDD have been closely associated with variations in circadian clock genes. Serretti et al. identified a significant link between the C allele of the single nucleotide polymorphism (SNP) rs1801260 on the *CLOCK* gene and initial insomnia; however, no correlation was found with middle insomnia, late insomnia, or hypersomnia [45]. This finding suggests that specific genetic variations may influence different types of sleep disturbances in distinct ways. Additionally, Antypa et al. reported that the CC genotype of the *CLOCK* gene is associated with altered sleep patterns in Caucasian women, highlighting potential gender and ethnic differences in the relationship between genetic variation and sleep disorders [46]. The *TIMELESS* gene plays a crucial role in the core feedback loop that regulates circadian rhythms. It interacts with CLOCK and BMAL1, affecting the stability and activity of these transcription factors and, consequently, modulating downstream gene expression. Notably, rare polymorphisms at the rs1082214 locus of the *TIMELESS* gene have been linked to sleep disturbances in males with depression. Among males with MDD who experience early morning awakenings, the minor T allele was significantly more common than in controls. Additionally, the rs7486220–rs1082214 GT haplotype showed a strong association with sleep disturbances [47], highlighting the role of specific genetic combinations in the pathogenesis of these disorders.

Additional circadian clock genes, such as CRY1, NFIL3, and RORC, have also been implicated in sleep-wake disturbances. Mutations in these genes are associated with an increased risk of MDD, particularly among individuals carrying mutations that disrupt physiological rhythms [48,49]. Studying these clock genes not only deepens our understanding of the biological foundations of sleep disturbances and MDD but also offers valuable insights for creating novel prevention and treatment strategies that are tailored to genetic predispositions.

## Diurnal mood variation and seasonal fluctuations in MDD

A key characteristic of circadian rhythm disruption in MDD is diurnal mood variation, often summarized as “morning low and evening improvement”. Patients typically experience their lowest mood in the morning, with a gradual improvement as the day progresses. This pattern is an important diagnostic indicator of MDD. The underlying mechanism may involve dysregulation of the hypothalamus-pituitary-adrenal (HPA) axis, which can lead to changes in the circadian rhythm of cortisol secretion. Normally, cortisol levels rise within the first hour after waking, then sharply decline over the next three hours, continuing to decrease throughout the day until reaching a low point during early sleep. During sleep, cortisol levels remain low and begin to rise again upon waking [50,51]. However, in MDD patients, the cortisol peak may occur 1–2 h earlier, resulting in a blunted morning rise and impaired nocturnal suppression of cortisol secretion This leads to elevated and flattened cortisol levels throughout the 24-h period, with insufficient decline during the night when cortisol should be at its lowest [52], which may contribute to the pronounced morning depressive symptoms. Dysregulated cortisol rhythms have been proposed as a biomarker for MDD, offering potential utility in diagnosis, therapeutic monitoring, and relapse prediction [53].

## Seasonal affective disorder (SAD)

Seasonal Affective Disorder, a subtype of major depressive disorder, occurs in a sizable minority of those with MDD. It is marked by predictable episodes of depression occurring in the autumn and winter months, with spontaneous improvement in spring and summer [54]. In the United States, about 5% of adults are affected by SAD, experiencing functional impairment for around five months each year [55]. Individuals with SAD often exhibit delayed sleep patterns and report hypersomnia. These seasonal depressive episodes are closely linked to decreased natural light exposure during the winter months. Additionally, reduced daylight exposure due to limited outdoor activity in modern lifestyles may further contribute to SAD susceptibility by disrupting normal circadian entrainment [56]. In healthy individuals, exposure to daylight helps synchronize the circadian clock through intrinsically photosensitive retinal ganglion cells, which regulate sleep, alertness, and mood [22]. For those with SAD, the reduction in daylight during winter may worsen mood dysregulation.

The relationship between SAD and melatonin, a hormone produced by the pineal gland, is a crucial area of research. Melatonin binds to receptors in the brain, activating enzymes that synthesize γ-aminobutyric acid (GABA) and increasing GABA levels in the hypothalamus, which promotes sleep. In healthy individuals, melatonin secretion rises before their usual bedtime, peaks around 3 am, and then declines to nearly undetectable levels during wakefulness. However, in patients with SAD, the normal rhythms of melatonin secretion may be disrupted, resulting in phase advances, phase delays, or reduced secretion levels. These changes are thought to contribute to the symptoms of SAD [57]. Research indicates light therapy demonstrates antidepressant efficacy in treating SAD, while melatonin supplementation may help regulate circadian phase and improve sleep quality SAD [58], these interventions may work by realigning or delaying circadian rhythms, correcting misalignment, and indirectly improving mood. The treatment of SAD typically involves targeted approaches, such as light therapy, whereas other forms of MDD may require pharmacological, psychological, or multimodal interventions. Accurately identifying SAD and addressing its specific mechanisms are essential for optimizing patient outcomes. Personalized treatment plans can more effectively alleviate symptoms and enhance quality of life for individuals with SAD.

## Circadian rhythms in eating behavior

The interaction between the circadian rhythm system and eating behavior is multifaceted, involving coordination between the central clock located in the SCN and peripheral clocks in other organs [21]. The SCN regulates eating rhythms by controlling the circadian secretion of appetite-related hormones, including leptin, orexin, and ghrelin [59]. Studies have shown significant heterogeneity in appetite regulation among MDD patients: approximately 48% of adults with MDD experience depression-associated appetite suppression, while around 35% report depression-related appetite increases [60]. Additionally, eating patterns are closely linked to MDD [61]. Night Eating Syndrome (NES) is a distinct eating disorder marked by a delayed eating rhythm that occurs during the evening and night, often accompanied by mood deterioration and depressive symptoms at specific times [62]. Notably, individuals with NES experience mood declines in the evening and night, which contrasts with the typical diurnal pattern of MDD, where mood tends to be lowest in the morning [63]. Additionally, the severity of self-reported depressive symptoms is significantly linked to NES. In mouse models of NES [64], depressive-like behaviors were observed during the light phase (which corresponds to the human evening and night) when feeding was restricted to the inactive period, even when the diet was normal in fat content [65]. These findings highlight the crucial role of the circadian rhythm system in regulating eating behavior and mood, as well as the intricate relationship between circadian rhythm disruptions and eating disorders like NES. Understanding these interactions provides valuable insights for developing targeted treatments for eating disorders and associated mood disturbances.

## Circadian rhythm alterations in metabolism

The circadian rhythm system is essential for maintaining human health and regulating metabolism. This system operates on a roughly 24-h cycle, coordinating and optimizing various physiological processes such as hormone secretion and energy metabolism. Patients with MDD frequently exhibit metabolic abnormalities, including weight changes, impaired glucose regulation, and dyslipidemia, which may be partly attributed to disturbances in their circadian rhythms. Clinically, MDD is often linked with metabolic syndrome (MetS) a cluster of conditions that encompasses obesity, hyperglycemia, insulin resistance, dyslipidemia, and hypertension. These conditions collectively heighten the risk of cardiovascular diseases and type 2 diabetes [66]. Research indicates that individuals with MetS exhibit higher levels of anxiety and depressive symptoms [67]. The interplay between these two conditions suggests overlapping pathological mechanisms between MDD and MetS [68]^,^ potentially involving multifaceted interactions such as inflammation, neurotransmitter systems, insulin resistance, lifestyle factors, and psychosocial elements [69,70]. A growing body of evidence supports the association between circadian rhythm disruptions, metabolic dysregulation, and MDD.

**Figure 3.**
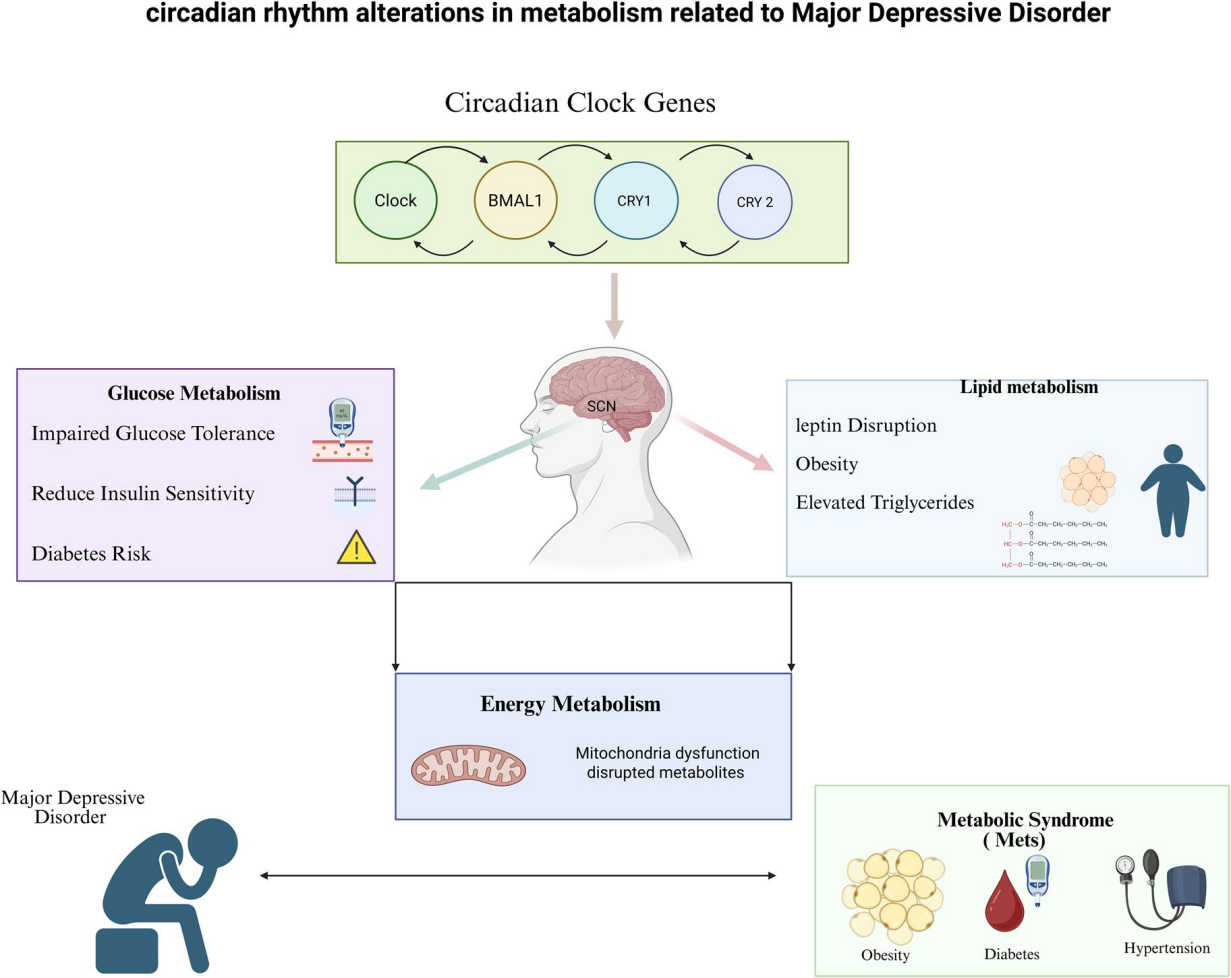
Circadian-metabolic crosstalk in major depressive disorder: from clock genes to metabolic syndrome. A. Core clock genes (CLOCK, BMAL1, CRY1/2) gate daily metabolic cycles. B. Their disruption in MDD drives glucose intolerance, leptin imbalance, and lipid dysregulation. C. Resulting mitochondrial dysfunction fuels obesity, insulin resistance, and progression to full metabolic syndrome.

Compared to the general population, individuals with MDD are more likely to exhibit obesity or dyslipidemia [70]. Circadian rhythms profoundly influence metabolism, particularly through the autonomic pathways of the SCN, which regulate lipid metabolism [71]. Sympathetic and parasympathetic neurons in the SCN play critical roles in controlling metabolic activities in adipose tissue, with sympathetic activation promoting lipolysis [72], and parasympathetic regulation governing anabolic processes in white adipose tissue [73]. Circadian rhythm disturbances often result in obesity and dyslipidemia. Studies have reported higher BMI and triglyceride levels in MDD patients with comorbid insomnia [74], highlighting the close link between circadian rhythms, obesity, and hyperlipidemia. Further investigations have revealed that the circadian clock gene Bmal1 regulates obesity and body weight in the hypothalamic SCN, with adipocyte-specific deletion of Bmal1 leading to obesity [75,76]. Leptin, a hormone with circadian rhythm properties, is critical for indicating obesity risk. [77]. Under normal conditions, leptin levels are low during the day and peak at night, facilitating proper appetite regulation and energy expenditure. However, disruptions in leptin rhythms, such as insufficient daytime levels or a lack of nighttime elevation, increase the risk of overeating and obesity development [78]. Studies in MDD patients have shown that leptin levels correlate with appetite and weight proportions, with elevated leptin levels observed in obese individuals due to increased body fat [79]. Treatment strategies for MDD should therefore consider appetite and weight management alongside interventions to restore circadian rhythms and alleviate metabolic disorders Figure 3.

The prevalence of MDD among diabetes patients is double that of the general population and is linked to poorer health outcomes [80]. Research indicates that sleep disturbances can impair glucose tolerance and elevate diabetes risk [81]. Additionally, studies have shown that **BMAL1** deficiency disrupts tissue glucose metabolism and systemic glucose homeostasis, which can lead to diabetes due to decreased insulin sensitivity and impaired glucose oxidation [82]. In healthy individuals, blood insulin levels, pancreatic β-cell glucose sensitivity, and skeletal muscle insulin sensitivity display circadian variations. However, disruptions in circadian rhythm can lower insulin sensitivity, thereby increasing the risk of diabetes [83–85]. Glucose tolerance also demonstrates circadian fluctuations, with healthy adults experiencing worse glycemic control in the evening and nighttime [86]. During these circadian disruptions, glucose metabolism is further impaired, increasing susceptibility to diabetes.

MDD patients frequently exhibit impaired energy metabolism, which is closely tied to mitochondrial dysfunction [87]. Maintaining normal circadian rhythms is essential for healthy energy metabolism. Clock genes such as **CLOCK**, **BMAL1**, **CRY1**, and **CRY2** are vital for metabolic regulation [88]. Disruption of these genes leads to a loss of metabolic rhythms and exacerbates metabolic disorders. For example, the loss of the **BMAL1** gene affects carbohydrate metabolism and alters fatty acid and amino acid metabolites [89]. Tissue-specific deletion of **BMAL1** also results in reduced metabolic rates, impaired triglyceride biosynthesis, and amino acid accumulation in mice [90]. Additionally, metabolism itself can regulate circadian rhythms, with the pentose phosphate pathway serving as a key regulator of oxidative and transcriptional oscillations. The production of nicotinamide adenine dinucleotide phosphate (NADPH) by this pathway is crucial for maintaining circadian rhythms [91]. In animal models with depression-like phenotypes, metabolites such as amino acids, lipids, hormones, and neurotransmitters in the blood also exhibit circadian rhythmicity [92,93]^.^ These findings underscore that disruptions in circadian rhythms affect not only sleep quality and emotional states but also metabolic processes, exacerbating depressive symptoms and contributing to the development of metabolic diseases.

## Alterations in the circadian rhythm of body temperature

Peripheral body temperature regulation is a complex physiological process that balances vasoconstriction, mediated by the sympathetic nervous system, and vasodilation in peripheral skin vessels, governed by the parasympathetic nervous system [94]. This balance is managed by the SCN, which regulates sympathetic and parasympathetic inputs to various organs to establish daily physiological rhythms [95]. In healthy individuals, peripheral body temperature naturally oscillates, rising in the morning and declining at night [96], reflecting the physiological process of central heat dissipation. However, this rhythm is often disrupted in patients with MDD [97], leading to flattened circadian rhythms, a lack of nighttime temperature decline, phase delays, and persistently low average temperatures [98], which may be linked to autonomic dysfunction. Research indicates that peripheral body temperature correlates with the severity of depressive symptoms and insomnia in MDD patients, while abnormal phase delays in temperature rhythms may be associated with an increased risk of suicide [99,100]. Conversely, reductions in peripheral temperature at night and the restoration of temperature rhythms are often associated with the alleviation of depressive symptoms [101,102], suggesting their potential as indicators of treatment efficacy. These findings underscore the critical role of body temperature regulation in maintaining homeostasis and highlight its importance as a marker for assessing MDD severity and a non-invasive biomarker for monitoring treatment outcomes.

## Alleviating depressive symptoms by improving circadian rhythms

Disturbances in circadian rhythms are closely linked to the development, symptoms, and progression of MDD. Regulating and restoring normal circadian rhythms may offer a promising therapeutic strategy for MDD. Research has shown that interventions such as light therapy, sleep deprivation, sleep-wake phase advancement, and pharmacological treatments targeting clock genes and related molecular pathways can improve both circadian rhythms and depressive symptoms. Future studies should explore the underlying mechanisms of these connections to develop more effective prevention and treatment methods for MDD.

## Pharmacological interventions

In recent years, there has been an increasing emphasis on the changes in the melatonergic system associated with MDD. Melatonin, a hormone produced by the pineal gland, plays a crucial role in regulating the sleep-wake cycle and circadian rhythms. Its secretion follows distinct circadian patterns, typically peaking at night to facilitate sleep. Melatonin and its analogs, including ramelteon, agomelatine, TIK-301, Neu-P11, and tasimelteon, have been studied for their potential in treating various sleep disorders and in resynchronizing disrupted circadian rhythms [103].

## Melatonin and melatonergic receptors in depression

Melatonin (N-acetyl-5-methoxytryptamine) is a neurohormone synthesized primarily by the pineal gland in a circadian manner, with high levels during the night and low levels during the day. It plays a crucial role in regulating circadian rhythms, sleep-wake cycles, and seasonal adaptations through activation of two G protein-coupled receptors, MT1 and MT2 [104,105]. These receptors are densely expressed in the SCN and other brain regions involved in mood regulation. Alterations in melatonin secretion and receptor expression have been consistently implicated in depression. Post-mortem studies have demonstrated increased MT1 receptor immunoreactivity in the SCN of depressed patients compared to controls, suggesting upregulation of melatonin signaling in depression [104]. The MT1 receptor appears particularly important, as genetic deletion of MT1 in mice leads to increased depressive-like behaviors in forced swim tests [105]. Both MT1 and MT2 receptors modulate neurotransmitter systems, neuroplasticity, and inflammatory pathways that are dysregulated in depression. Melatonin receptor agonists such as agomelatine, ramelteon, and tasimelteon have been developed for treating depression and circadian rhythm disorders. Agomelatine, a dual MT1/MT2 agonist and 5-HT2C antagonist, has demonstrated antidepressant efficacy comparable to SSRIs while also improving sleep quality [106]. These agents work by resynchronizing circadian rhythms, enhancing neurogenesis, and modulating monoaminergic neurotransmission.

Melatonin regulates circadian rhythms by interacting with melatonin receptors (MT1 and MT2), which play a crucial role in its regulatory functions [107,105]. Additionally, these receptors are linked to the modulation of anhedonia and anxiety-like behaviors [105]. Animal studies have shown that melatonin exhibits antidepressant and anti-stress effects, demonstrating its capacity to alleviate depressive-like behaviors caused by chronic stress [108], and to prevent depressive responses to acute stress [109]. These benefits are primarily attributed to the activation of melatonin receptors and their interactions with GABAergic, serotonergic [110] and glutamatergic systems, as well as the hypothalamic-pituitary-adrenal (HPA) axis [111]. However, current clinical studies have yet to establish conclusive evidence for the direct antidepressant effects of melatonin in humans. Most clinical research has focused on utilizing melatonin to alleviate sleep disturbances associated with depressive symptoms rather than targeting depressive disorders directly.

Agomelatine is a novel compound that integrates non-monoaminergic signaling with traditional monoaminergic mechanisms [112]. It activates human MT1 and MT2 receptors [106,113], mimicking melatonin’s effects by regulating circadian rhythms through the suppression of SCN neuronal firing rates. Additionally, agomelatine acts as a 5-HT2C antagonist, contributing to the regulation of mood and stress, synchronization of circadian rhythms, and improvement of sleep quality [114]. Research suggests that its antidepressant effects arise from the interaction between its melatonergic agonism and 5-HT2C antagonism [115]. Clinical studies indicate that agomelatine significantly alleviates depressive symptoms, particularly in patients with anxiety-related depression, and its effects are superior to those of placebos and other antidepressants [116]. Moreover, agomelatine has demonstrated specific efficacy in alleviating anxiety symptoms among patients with major depression, suggesting a broad psychotropic profile beyond mood regulation [117]. The antidepressant properties of agomelatine are also associated with reductions in circulating C-reactive protein levels in patients with MDD who achieve remission, indicating potential anti-inflammatory effects [118]. These findings highlight the potential of melatonin and agomelatine in regulating circadian rhythms and alleviating depressive symptoms, especially in the management of sleep and anxiety disorders. However, further research is necessary to clarify their mechanisms and establish their definitive roles in treating MDD.

Circadian rhythm oscillations influence the pharmacokinetics and pharmacodynamics of medications, affecting absorption, distribution, metabolism, and elimination, along with interactions with intracellular signaling, target molecules (such as receptors, transporters, and enzymes), and gene transcription [119]. Thus, optimizing dosing schedules to account for these circadian influences is essential for enhancing therapeutic efficacy, minimizing side effects, and improving patient adherence and quality of life.

## Chronotherapeutic Approaches

Chronotherapeutic is a treatment approach based on chronobiological principles, leveraging the rhythmic variations of the body’s internal biological clock to optimize therapeutic outcomes. It is particularly effective in the management of MDD. The main components of chronotherapeutic include sleep deprivation (SD), sleep phase advance (SPA), and light therapy (LT) [120].

### Sleep deprivation (SD)

SD involves intentionally maintaining wakefulness to disrupt the patient’s sleep pattern temporarily, aiming to provide rapid relief from depressive symptoms [121]. Despite its rapid antidepressant effects, SD must be administered with caution. Potential side effects include the induction of manic switches in vulnerable individuals, increased anxiety, and in some cases, panic attacks or rapid relapse upon the next sleep episode.

### Light therapy (LT)

LT employs morning light exposure to regulate the circadian clock and correct disruptions in diurnal rhythms, often used to treat seasonal affective disorder (SAD). While originally developed for SAD, LT has also demonstrated efficacy in treating non-seasonal major depressive disorder, either as monotherapy or as an adjunct to conventional antidepressant treatment [122].

Studies have shown that combining SPA and LT with SD referred to as “adjunctive triple chronotherapy” effectively alleviates depressive symptoms and helps prevent relapse following SD [123,124].Triple chronotherapy stabilizes circadian rhythms and shows 50–84% response rates for depression with minimal side effects, making it a safe and effective add-on treatment for unipolar and bipolar depression [125]. This integrated approach enhances the efficacy of conventional antidepressants, and extends therapeutic benefits for approximately nine weeks [123]. Furthermore, it is considered feasible and well-tolerated for patients with MDD who are at acute suicidal risk [126]. The effectiveness of chronotherapeutic is likely linked to their ability to regulate circadian rhythms. For instance, prolonged wakefulness during SD can increase cortical excitability, glutamate release, and synaptic strength contributing to mood improvement in MDD patients. Additionally, SD may enhance the circadian rhythmicity of clock gene expression mechanisms [127]. A recent transcriptomic study involving 78 SD patients supported this hypothesis, revealing increased expression of circadian-related genes, such as **PER1**, in responders compared to non-responders [128]. “Adjunctive triple chronotherapy” serves as an effective supplement to traditional antidepressant treatments, offering a more comprehensive therapeutic strategy for MDD patients. However, it is important to note that this approach is effective in only 40-60% of MDD patients [129]. Non-responders may represent a subgroup without circadian rhythm abnormalities associated with depression.

## Dark therapy and dynamic lighting

Beyond traditional light therapy, “virtual dark therapy” using blue-blocking (BB) glasses has emerged as a promising intervention. By filtering short-wavelength light in the evening, BB glasses prevent the suppression of melatonin and help stabilize the circadian phase, particularly in patients with sleep-onset insomnia [130]. Additionally, dynamic lighting systems that mimic the natural progression of daylight intensity and color temperature have shown efficacy in inpatient settings, improving sleep continuity and mood stability [131].

## Cognitive behavioral therapy and interpersonal and social rhythm therapy

Cognitive Behavioral Therapy (CBT) and Interpersonal and Social Rhythm Therapy (IPSRT) are evidence-based psychotherapeutic approaches that effectively improve depressive symptoms by reducing negative affect, stabilizing mood, and enhancing emotional regulation in individuals with depression.

### Cognitive behavioral therapy (CBT)

CBT focuses on identifying and altering negative thought patterns and maladaptive behaviors. A specialized branch, Cognitive Behavioral Therapy for Insomnia (CBT-I), targets sleep-related issues by educating patients on good sleep hygiene, relaxation techniques, stimulus control, and sleep restriction strategies [132]. Research indicates that CBT-I not only improves sleep but also alleviates depressive symptoms [133]. Digital CBT-I, delivered *via* electronic devices and the internet, has similarly proven effective in addressing insomnia and reducing depressive symptoms [134]. Recent studies suggest that combining digital CBT-I with measures to enhance circadian support such as regular bright light exposure, physical activity, and body temperature regulation significantly lowers depression risk in insomnia patients [135]. This comprehensive approach has also been shown to enhance amygdala responsiveness [136], suggesting deeper impacts on emotional regulation.

### Interpersonal and social rhythm therapy (IPSRT)

IPSRT is tailored for individuals with bipolar disorder (BD) and aims to stabilize circadian rhythms while applying interpersonal psychotherapy techniques to assist patients in managing emotional challenges. A one-year study revealed that IPSRT not only significantly reduces depressive symptoms but also decreases hospitalization rates and lowers the risk of recurring manic and depressive episodes [137]. Additionally, for older outpatient populations, whether diagnosed with MDD or BD, group IPSRT sessions held twice a week have proven to be both feasible and well-received [138].

CBT and IPSRT offer effective psychotherapeutic options for MDD patients, addressing emotional dysregulation through distinct mechanisms. CBT-I stands out for its success in treating insomnia, while IPSRT shows promise in stabilizing mood and reducing hospitalizations. Further research and implementation of these therapies could provide more comprehensive support for individuals with mood disorders.

## Other therapies

Emerging studies have revealed that the gut microbiome and its metabolites exhibit circadian rhythmicity, primarily influenced by feeding and fasting cycles. Persistent jet lag, obesogenic diets, and clock gene deficiencies can suppress bacterial rhythmicity in the gut [139]. Probiotic supplementation has been found to improve both sleep quality and depressive symptoms [140]. Additionally, thermoregulatory interventions, such as hot water baths, saunas, or whole-body hyperthermia, have shown potential for alleviating depressive symptoms. These methods are safe, easy to implement, and can be incorporated into comprehensive treatment plans for MDD [141].

## Limitations

This study has several limitations. A fully systematic search strategy and formal risk of bias assessment were not conducted, which may limit reproducibility and introduce potential selection bias. Therefore, some interpretations should be considered in light of the quality and consistency of the available evidence.

## Conclusion

Circadian rhythm disruption is closely linked to the pathophysiology of major depressive disorder, influencing sleep, mood regulation, metabolic function, and neurobiological processes. Understanding these mechanisms may improve the clinical management of MDD through more targeted circadian-informed interventions.

### Road to future

The interaction between circadian rhythms and major depressive disorder is increasingly recognized as central to disease mechanisms. Future research integrating genomics, biological timing, and lifestyle factors may improve early diagnosis and enable more precise and effective treatments. Continued interdisciplinary efforts are expected to translate these advances into better clinical outcomes and quality of life for patients.

## Data Availability

Data sharing not applicable – Data sharing is not applicable to this article as no new data were created or analyzed in this research.

## Open scholarship

This article has earned the Center for Open Science badges for Open Materials. The materials are openly accessible at 10.6084/m9.figshare.32041353.
